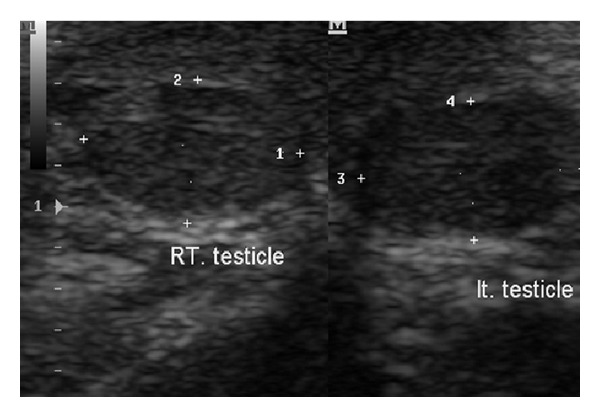# Idiopathic Scrotal Hematoma in Neonate: A Case Report and Review of the Literature

**DOI:** 10.1155/2014/212914

**Published:** 2014-05-27

**Authors:** Bioku Muftau Jimoh, Eziechila Bessie Chinwe, Adewumi Oluwafemi Adebisi, Igwilo Chinwe Ifeoma, Maduka Ogechi, Aiyekomogbon Joshua Oluwafemi

**Affiliations:** ^1^Department of Surgery, Urology Division, Federal Staff Medical Centre, Abuja, Nigeria; ^2^Department of Pediatrics, Federal Staff Medical Centre, Abuja, Nigeria; ^3^Department of Obstetrics and Gynecology, Federal Staff Medical Centre, Abuja, Nigeria; ^4^Department of Radiology, Federal Staff Medical Centre, Abuja, Nigeria

## Abstract

Neonatal scrotal hematoma is a rare genitourinary emergency. Some cases have underlining aetiologic factors such as testicular torsion, adrenal hemorrhage, or birth trauma, and others are idiopathic. Previously, immediate scrotal exploration was considered imperative for diagnosis and treatment. With good imaging techniques, some patients are managed nonoperatively. We report a case of idiopathic scrotal hematoma in a neonate. He was managed conservatively with clinical and radiological follow-up. There was complete resolution of hematoma within two months, thus, avoiding unnecessary exploration.

## 1. Introduction

Scrotal hematoma in the neonate, though a rare condition, warrants prompt diagnosis and urgent intervention. It commonly results from testicular torsion, adrenal hemorrhage, and birth trauma.

However, in some cases no cause may be discernable. Since Putnam recorded his first experience in 1989 [1], few other cases of neonatal scrotal hematoma have been documented in the literature. We report a case of idiopathic left hemiscrotal hematoma in a newborn which is managed nonoperatively in our hospital. It reechoes the significance of neonatal scrotal and abdominal ultrasonography, thus preventing unnecessary scrotal exploration.

## 2. Case Presentation

A term male neonate with vertex presentation was delivered spontaneously per vaginuum to a 45-year-old P_7_^+3^ seamstress in a maternity home. The birth weight was 3 kg. Antenatal care was inadequate. Painless left hemiscrotal swelling was noticed 72 hours after birth by the pediatricians managing him for neonatal jaundice and sepsis. There was no bleeding from any body orifice.

**Figure 1:**
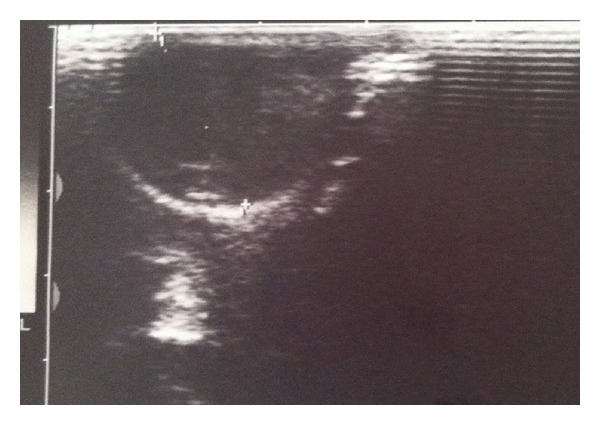


There was no bruise over the groin. Abdominal examination was normal. External genitalia revealed scrotal asymmetry with a fluctuant, nontender, left hemiscrotal swelling. The ipsilateral testis could not be palpated separate from the swelling. The swelling did not transilluminate and we could get above it. There was no evidence of trauma. An initial suspicion of acute scrotum was made. An emergency scrotal ultrasonography showed both testes were normal in their positions, sizes, and outline. The left tunica revealed fluid collection with high level echoes (Figure 1). The testicles showed normal parenchyma echo pattern and normal blood flow on color Doppler interrogation. The testicular arteries were also preserved.

The abdominal ultrasonography was normal. His packed cell volume was 42.6%, and clotting profile and platelet count were within normal limits. Direct bilirubin dropped from 15.2 mg/dL to 12.8 mg/dL after 4 days of phototherapy. The sepsis was treated with antibiotics. He had parenteral vitamin K. The Direct Coombs test was negative. A diagnosis of idiopathic left hemiscrotal hematoma was entertained. The child was managed nonoperatively. He was monitored clinically and radiologically. At two- and ten-month clinic follow-up, the patient was well and weighed 6.9 kg and 9.3 kg, respectively. Follow-up ultrasounds scan revealed complete resolution of the earlier noted left scrotal hematoma. The testicular parenchyma echo pattern was also preserved bilaterally (Figure 2).

## 3. Discussion

Acute scrotal hematoma in the neonate is an urgent genitourinary condition. Though its list of differential diagnoses is legion, it is necessary to rule out torsion of spermatic cord which truly warrants emergency surgical exploration [2]. Other common aetiologies of neonatal hemoscrotum include adrenal hemorrhage and birth trauma.

Spontaneous idiopathic hemorrhage of the scrotum, as found in our patient, has also been described [3–5]. This is usually a diagnosis of exclusion but uncommonly encountered.

Our patient presented with hemiscrotal swelling which was misdiagnosed as congenital hydrocele. The swelling, however, did not transilluminate as would be ordinarily expected in this age group. There was no clinical feature suggestive of trauma or bleeding diathesis. Scrotal and abdominal ultrasonography excluded the common aetiologies of neonatal hemoscrotum.

In most parts of our region where ultrasonography is not easily accessible and affordable, it is better to explore the scrotum so as to save the gonad (testis) in case the cause is testicular torsion [6]. However, in areas where ultrasound facilities abound, the imaging modality may be diagnostic or rule out testicular torsion. The contemporary colour Doppler sonography often demonstrates the intrascrotal anatomy and perfusion as well as testicular blood flow [7–10].

Also, radioisotope testicular scanning may be useful in assessing testicular perfusion with high specificity and sensitivity for testicular torsion [10, 11].

Few cases of adrenal hemorrhage induced hemoscrotum have been reported in the literature [1, 8, 12]. The condition could be spontaneous or may follow difficult or traumatic delivery [8].

In neonates, the suprarenal glands are relatively big and are vulnerable to vascular injury [13, 14]. The resultant hemorrhage is contained within the adrenal capsule. However, the capsule may rupture and the blood spreads to retroperitoneum or peritoneal cavity [15]. From the latter, the blood tracks down the scrotum via the inguinal canal with the patient presenting with bluish discoloration that extends from groin to the scrotum. In this case, scrotal and abdominal ultrasonography must be considered but imaging tool rarely distinguishes between adrenal hemorrhage and other causes of suprarenal masses [12, 14, 16].

Except for spermatic cord torsion, hemoscrotum from other causes may be managed nonoperatively. We treated our patient as such. He was monitored clinically and radiologically with good outcome. This modality was documented by other workers [8]. Surgical exploration is only indicated if conservative protocol fails or if the hematoma becomes infected.

## 4. Conclusion

Neonatal hemoscrotum is a condition rarely seen in surgical practice. When encountered it is important to speedily exclude testicular torsion which truly warrants emergency scrotal exploration. Urgent scrotal and abdominal ultrasonography is the diagnostic modality of choice. This may prevent unnecessary scrotal exploration.

## Figures and Tables

**Figure 2:**